# Human Stem Cell Transplantation, Immunosuppression, and Noninvasive *In vivo* Cell Tracking in the Mouse Brain

**DOI:** 10.1002/cpz1.70442

**Published:** 2026-08-22

**Authors:** Kevin S. Chen, Kyle J. Loi, Lisa M. McGinley, Osama N. Kashlan, Diana M. Rigan, Shayna N. Mason, Jacquelin F. Kwentus, Andrew D. Carter, Masha G. Savelieff, Eva L. Feldman

**Affiliations:** ^1^ Department of Neurology University of Michigan Ann Arbor MI USA; ^2^ NeuroNetwork for Emerging Therapies University of Michigan Ann Arbor MI USA; ^3^ Department of Neurosurgery University of Michigan Ann Arbor MI USA; ^4^ Department of Biomedical Sciences University of North Dakota Grand Forks ND USA

**Keywords:** Alzheimer's disease, bioluminescence imaging, fimbria fornix, hippocampus, immunocompetent model, stereotactic surgery

## Abstract

Stem cell therapies have emerged as potential therapeutics of interest for many central nervous system diseases, including stroke, epilepsy, neurodegenerative diseases, and traumatic brain injury. Preclinical models are required to evaluate the migration of transplanted stem cells to various areas of the brain, monitor graft survival to improve immunosuppression regimens, and assess stem cell safety and their efficacy on disease outcomes. Understanding all these aspects of stem cells as therapeutics can help optimize parameters for translation to humans. To facilitate this overarching goal, we report a protocol for human stem cell transplantation into mouse fimbria fornix, the outflow white matter tract of the hippocampus, which facilitates stem cell dissemination to regions distant to the injection site. Although our protocol involves injecting human neural stem cells expressing insulin‐like growth factor 1 into an Alzheimer's disease mouse model, it can be altered for application to diverse stem cells and cellular therapeutics, secreting various trophic factors, at multiple dosing schemes, and in different central nervous system targets and disorder models. Our approach is robust and highly reproducible, with demonstrated success in improving memory performance in an Alzheimer's disease mouse model and achieving long‐term survival of transplanted cells (up to 32 weeks) in immunocompetent mice, facilitating stem cell studies in the context of neuroimmune crosstalk. Our primary protocol involves administering an anti‐CD4/anti‐CD40L monoclonal antibody cocktail to prevent graft rejection, with bioluminescence imaging to track transplanted stem cells *in vivo*. We also present an alternate immunosuppression protocol that relies on tacrolimus and mycophenolate mofetil, which is less costly, albeit less effective (especially in the longer term), than the CD4‐CD40L antibody regimen. © 2026 The Author(s). *Current Protocols* published by Wiley Periodicals LLC.

**Basic Protocol**: Human stem cell transplantation into mouse hippocampus, antibody‐based immunosuppression, and *in vivo* tracking by bioluminescence imaging

**Alternate Protocol**: Immunosuppression by tacrolimus and mycophenolate mofetil

## INTRODUCTION

Disorders involving the central nervous system (CNS) collectively are among the top causes of death and disability across the globe (Ding et al., 2022; GBD 2021 Nervous System Disorders Collaborators, 2024). These include stroke, epilepsy, neurodegenerative diseases, congenital disorders, and traumatic injuries. Despite steady progress across all fields, cost‐effective, disease‐modifying therapies remain lacking for the vast majority of these conditions. This dearth of therapies can in part be attributed to the multifactorial pathophysiology of these neurological conditions and the difficulty of restoring neuroglial populations and connections in adults. Through their salutary pleiotropic effects, stem cell therapies have emerged as a potential strategy to combat CNS damage (Bonilla & Zurita, 2021; Chen et al., 2024). Stem cells can support injured neurons by secreting neurotrophic factors, exerting immunomodulatory actions, and scavenging toxins, generating a CNS milieu that nurtures and heals neural tissues (Chen et al., 2024). Stem cells can also differentiate into neurons, forging new synapses and integrating into and rebuilding existing neural networks, or into supportive glia, promoting overall repair or replacement of injured neural tissues. Indeed, translational research to restore neural function is well underway (Espuny‐Camacho et al., 2018; Kitahara et al., 2020; Revah et al., 2022; Tornero et al., 2013).

Nevertheless, despite this promise, several barriers hinder stem cell advances, among them the challenges posed by the blood‐brain barrier, which obstructs delivery of biologics, including cellular therapeutics, into the CNS (Chen et al., 2024). Additionally, verifying and enhancing graft survival is crucial for maximizing the lifespan of the cellular therapeutics and associated benefits. In xenograft animal models or allograft human clinical trials, immunosuppression appears to be necessary to optimize graft survival despite the traditionally held view of the CNS as an immune‐privileged organ (McGinley et al., 2022; Tadesse et al., 2014). To overcome these gaps, we developed a human stem cell transplantation method that directly delivers cellular therapeutics into the mouse brain, which can be noninvasively tracked *in vivo* to monitor graft dissemination and survival (Chen et al., 2023; McGinley et al., 2017; McGinley et al., 2018; McGinley et al. 2021; McGinley et al. 2022). Additionally, we established a monoclonal antibody (mAb)‐based immunosuppression regimen for human stem cell transplantation (McGinley et al., 2022) that targets T cell co‐stimulatory CD4 and CD40L, inhibiting graft rejection (Wood et al., 1996).

Our protocol has several preclinical and translational applications, including testing the safety and efficacy of stem cells or other cellular therapeutics for CNS disorders, assessing the dissemination of transplanted stem cells across brain regions, and evaluating graft survival to optimize therapeutic and immunosuppression approaches. Delivery of materials by intravenous, intra‐arterial, and intracisternal/subarachnoid methods has been described, and these are arguably less invasive, but we find that direct intracerebral injection maximizes cell dosage and regional anatomic specificity. Here, we report a protocol for human stem cell transplantation into mouse fimbria fornix, the outflow white matter tract of the hippocampus. However, the protocol can be adjusted to target different brain areas by using standard published brain atlases (Wang et al., 2020). Additionally, although this protocol is based on transplantation of human neural stem cells, our technique can be applied to precisely deliver diverse alternative biological payloads into mouse brain (e.g., other cell types, extracellular vesicles, viruses, nucleic acids, proteins, etc.).

The primary protocol leverages bioluminescence imaging (BLI) to track the transplanted stem cells *in vivo* and a CD4‐CD40L mAb regimen to prevent graft rejection. Although clinical stem cell protocols to date have relied on solid‐organ transplant experience (Feldman et al., 2014; Kondziolka et al., 2005), we found that using an mAb‐based immunosuppression regimen is critical for maximizing graft recovery while minimizing the anxiety and stress of daily handling and intraperitoneal injections of tacrolimus and mycophenolate mofetil immunosuppression, which may adversely affect behavioral readouts. Nevertheless, we do additionally provide an alternate, lower‐cost protocol of tacrolimus and mycophenolate mofetil immunosuppression, although this is less effective than our CD4‐CD40L antibody regimen in the longer term (i.e., >1 week duration; McGinley et al., 2022).

Overall, although stereotactic targeting in the rodent brain is well established, our protocol herein combines noninvasive tracking methods with reliable long‐term xenograft survival in an immunocompetent host. Thus, our approach is immensely useful for translational studies as well as for mechanistic studies of stem cell transplantation in the context of neuroimmune crosstalk in neurodegenerative diseases (Scheiblich et al., 2020) and neural repair (Shichita et al., 2023). Additionally, our protocol performs transplantation into the fimbria fornix, which, as the efferent and afferent white matter tracts of the hippocampus, facilitates the movement of a portion of stem cells to regions distant from the injection site.

*NOTE*: This entire protocol follows all appropriate institutional and U.S. national guidelines and regulations per the U.S. National Institutes for Health for using and handling human stem cells and mice. All experiments must be conducted according to Institutional Animal Care and Use Committee as well as Institutional Biosafety Committee‐approved protocols.

## STRATEGIC PLANNING

The Basic Protocol presents an optimized surgical technique for targeted injection of human stem cells into the fimbria fornix of the hippocampus—the white matter outflow tract from the hippocampus—in the mouse brain. Because we are interested in stem cell therapeutics for dementia, we perform transplants into transgenic Alzheimer's disease (AD) mouse models, such as the APP/PS1 or 5XFAD models. APP/PS1 mice harbor human amyloid‐beta precursor protein (*APP*) with K670N and M671L mutations and human presenilin‐1 (*PSEN1*) with M146L mutation and develop cognitive impairment within 3 to 6 months of age, amyloid plaques within 6 months, microgliosis within 9 to 12 months of age, and neurodegeneration within 22 months (Drummond & Wisniewski, 2017). By contrast, 5XFAD mice express five familial AD mutations, including the Swedish (K670N/M671L), Florida (I716V), and London (V717I) mutations in the human *APP* gene, as well as M146L and L286V mutations to human *PSEN1*. The 5XFAD mouse model develops the hallmarks of AD, with brain amyloid plaques and microgliosis by 2 months of age, cognitive impairment within 4 to 6 months, and neurodegeneration within 9 months (Drummond & Wisniewski, 2017). We transplant stem cells into 5XFAD mice of various ages, including as early as 8 to 10 weeks (McGinley et al., 2022) or as old as 26 weeks (Chen et al., 2023), and have transplanted cells into APP/PS1 mice at 12 weeks of age (McGinley et al., 2018). Therefore, our approach is amenable to different AD mouse models and may be compatible with a range of alternative models of neurological disease.

We have injected insulin‐like growth factor 1 (IGF‐1)‐expressing human neural stem cells (IGF1‐hNSCs), but the protocol is broadly applicable to various alternative stem cell or cellular therapeutics. The provenance, ethical considerations, and establishment of this IGF1‐hNSC line have been previously reported, and meet all consents recommended by the U.S. National Institutes of Health and the U.S. Food and Drug Administration, and the guidelines of an outside independent review board (Goutman et al., 2019; McGinley et al., 2016). IGF‐1 is a neurotrophic molecule that promotes synaptogenesis and neurogenesis; secreted from IGF1‐hNSCs, IGF‐1 protects primary cortical neurons from amyloid‐beta induced apoptosis (McGinley et al., 2016). The IGF1‐hNSCs were also modified to express firefly luciferase for *in vivo* tracking by BLI in live mice and green fluorescent protein (GFP) for immunohistochemical analysis at study end in brain tissue (McGinley et al., 2022). However, the protocol can be implemented for stem cells that secrete different bioactive molecules or reporter molecules, e.g., red fluorescent protein instead of GFP.

Before transplantations, we randomize disease model animals into untreated, vehicle‐injected (sham or vehicle), and cell‐injected groups, which are compared to age‐ and sex‐matched nontransgenic, untreated wild‐type (WT) littermate controls. When we initially developed and optimized our procedural approach, we included a sham/vehicle‐injected WT group and found that cognitive performance was unaffected; nevertheless, a sham/vehicle‐injected WT group should be considered when adapting and optimizing this protocol to novel situations. Because we implement xenograft transplantation of a human neural stem cell line into mice, control groups of 5XFAD and WT animals receiving immunosuppression alone have also previously been tested in our lab to rule out behavioral/biological effects of the immunosuppressant. Although we found no detrimental effect from the mAb immunosuppression regimen, utilizing this control in new experimental situations should be considered. Vehicle‐injected animals serve to assess the effects of the injection, carrier solution, and any other part of the surgical procedure on outcome measures as compared with the untreated disease model animals. Other experimental paradigms may require additional groups, such as injection of an inactivated therapeutic/dead cells to compare to the treatment group, or cell‐injected groups of stem cells with and without expression of neurotrophic or other factors. Additionally, two or more stem cell dosages may be assessed. Regarding attrition, in our hands, one mouse out of 15 dies shortly after surgery, i.e., between 2 and 10 days postoperative. Additionally, depending on the disease model and mouse age, further attrition may also occur. Thus, the number of mice per group will depend on the anticipated effect size as well as attrition due to surgical complications and disease progression.

In our experience, IGF1‐hNSC transplantation improves cognition in the spatial memory domain by Morris water maze 8 weeks post injection when applied to 26‐week‐old 5XFAD mice (Chen et al., 2023). IGF1‐hNSC transplantation also ameliorates cognition by novel object recognition test (4 weeks post injection) and Morris water maze (16 weeks post injection) when applied to 12‐week‐old APP/PS1 mice (McGinley et al., 2018). Analysis of brain tissue at study ends demonstrates graft survival as well as dissemination of IGF1‐hNSCs across the hippocampus, cortex, and other brain areas, although most cells remain within the white matter tracts of the fimbria fornix and corpus callosum. Ultimately, the appropriate time line and assays for evaluating treatment response will differ according to the expected effects of therapy and the animal model employed.

*NOTE*: All protocols involving animals must be reviewed and approved by the appropriate Animal Care and Use Committee and must follow regulations for the care and use of laboratory animals. Appropriate informed consent is necessary for obtaining and use of human study material.

## Basic Protocol 1 HUMAN STEM CELL TRANSPLANTATION INTO MOUSE HIPPOCAMPUS, ANTIBODY‐BASED IMMUNOSUPPRESSION, AND *IN VIVO* TRACKING BY BIOLUMINESCENCE IMAGING

**Figure 1:**
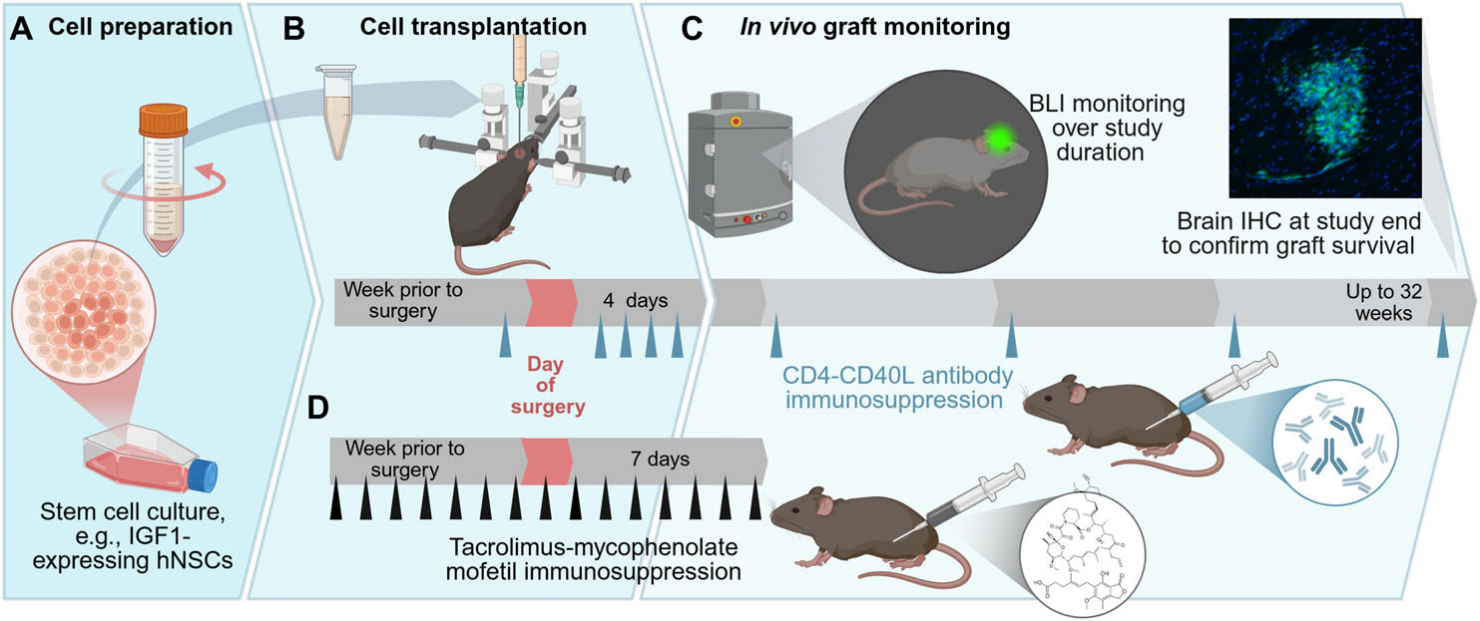
Overarching protocol process. (**A**) Section 1, “cell preparation,” emphasizes appropriate stem cell culturing and preparation for injection, e.g., insulin‐like growth factor 1‐expressing human neural stem cells (IGF1‐hNSCs). (**B**) Section 2, “cell transplantation,” focuses on the surgical procedure for injecting the IGF1‐hNSCs. Immunosuppressant administration begins 1 day before surgery for the CD4‐CD40L antibody regimen (teal triangles) and 1 week before surgery for the tacrolimus‐mycophenolate mofetil regimen (black triangles). (**C**) Section 3, “*in vivo* graft monitoring,” details continued postoperative administration of the immunosuppressant CD4‐CD40L antibody regimen, given weekly after the first four postoperative injections, with tandem *in vivo* graft monitoring by bioluminescence imaging for up to 32 weeks. During this phase, tests can assess the efficacy of IGF1‐hNSC transplantation for slowing or reversing disease progression. (**D**) An alternate protocol uses daily tacrolimus and mycophenolate mofetil for immunosuppression, which starts 1 week before surgery, with one injection on the day of surgery and 1 week of injections afterward.

In this protocol, we describe the transplantation of IGF1‐hNSCs into the fimbria fornix of the hippocampus of 5XFAD or APP/PS1 mice and the administration of an immunosuppression regimen to promote graft survival, which can be monitored *in vivo* (however, any stem cell line or biologic under investigation may be used instead, as needed). The overarching process can be divided into three sections (Fig. 1A–C). Section 1 emphasizes IGF1‐hNSC culturing and preparation for injection, whereas section 2 focuses on the surgical procedure for injecting the IGF1‐hNSCs. Section 3 details postoperative administration of an immunosuppression CD4‐CD40L antibody regimen with tandem *in vivo* graft monitoring by BLI. During this phase, tests can assess the efficacy of IGF1‐hNSC transplantation for slowing or reversing disease progression. We additionally provide an alternate protocol for immunosuppression with tacrolimus and mycophenolate mofetil (Fig. 1D; see Alternate Protocol).

Notably, this protocol may be adapted to other cell types and animal models. Cell‐ and animal‐specific differences will need to be considered (e.g., cell viability and culture conditions, injection targets, etc.). We have also provided a list of materials we commonly use; however, most supplies/equipment and reagents can be substituted depending on available manufacturers and local protocols.

### Materials

*Stem cell culture and preparation*:
Cell line: e.g., IGF1‐hNSC cell line (previously from Palisade Bio; now available upon request from the Feldman Laboratory at the University of Michigan), growing in T175 flask in growth medium supplemented with 10 ng/ml basic fibroblast growth factor (bFGF)
1× phosphate‐buffered saline (PBS; see recipe), prewarmed to 37°C
0.25% trypsin‐EDTA (Gibco, Thermo Fisher Scientific, cat. no. 25200056)
Soybean trypsin inhibitor (Gibco, cat. no. 17075029) dissolved to 0.5 mg/m in Dulbecco's phosphate‐buffered saline without calcium or magnesium (Gibco, cat. no. 14190144)
Hibernation medium (see recipe; carrier buffer for stem cells, designed to maximize survival)
0.4% trypan blue solution (Gibco, Thermo Fisher Scientific, cat. no. 15250061)
Poly‐d‐lysine‐coated T175 (175‐cm^3^) culture flasks (Nalgene Nunc, Thermo Fisher Scientific, cat. no. 132705)

Biosafety Level 2 cell culture hood
1‐, 5‐, 10‐, and 25‐ml sterile serological pipets (ThermoScientific Nunc, cat. nos. 170353N, 170355N, 170356N, and 170357N)
15‐ and 50‐ml sterile orange‐cap tubes (Corning, cat. nos. 430766 and 430291)
Incubator, 37°C, 5% CO_2_
2‐ml cryovials, sterile
P200 and P1000 (200‐ and 1000‐µl) micropipets
Sterile micropipet tips (Fisher Scientific, cat. nos. 02‐707‐421 [P200] and 02‐707‐406 [P1000])
General benchtop centrifuge (Sorvall, Thermo Fisher Scientific, cat. no. 75004241), swinging bucket rotor (Thermo Fisher Scientific, cat. no. 75003629), and tube holder (ThermoScientific, cat. no. 75003682 and 75003683)
Pipettor (Drummond, cat. no. DP‐102)
Hemocytometer (Hausser Scientific, Fisher Scientific, cat. no. 02‐671‐10)
Inverted light microscope (Nikon, cat. no. TS100)

*Animal procedure*:
5XFAD (Jackson Laboratory, cat. no. 034848) or APP/PS1 (Jackson Laboratory, cat. no. 034829) mice and WT littermate controls (alternative models using the same background strain should use injection coordinates presented here if the fimbria fornix is targeted; other strains, or animals injected before adulthood, may require testing and adjustment of injection coordinates)
CD4 antibody: clone GK1.5, rat IgG2b, *κ* (Bio X Cell, cat. no. BE0003‐1)
CD40L antibody: clone MR‐1, Armenian hamster IgG (Bio X Cell, cat. no. BE0017‐1)
70% (v/v) ethanol (70:30 [v/v] 200‐proof ethanol [Decon Labs, cat. no. 2701]/H_2_O)
Fluriso (isoflurane, USP grade; VetOne, cat. no. V1 501017, National Drug Code [NDC] 13985‐528‐40)
Oxygen, medical USP grade (Cryogenic Gases, cat. no. OXY‐USP‐EAL)
0.3 mg/ml buprenorphine hydrochloride (Par Pharmaceutical, NDC 42023‐179‐05)
Puralube Vet Ointment (sterile ocular lubricant; Dechra, NDC 17033‐211‐38)
Chlorhexidine, 4% (antiseptic, antimicrobial skin cleanser; McKesson, cat. no. 16‐CHG8, NDC 68599‐5401‐3)
Sterile water (for irrigation; Baxter Healthcare Corporation, cat. no. 2F7112, NDC 0338‐0004‐02), prewarmed to ~37°C
Sterile saline: 0.9% (w/v) sodium chloride (for irrigation; Baxter Healthcare, cat. no. 2F7122, NDC 0338‐0048‐04)
50 mg/ml carprofen (Zoetis, NDC 54771‐8507‐1)

Central Line Pack (Medline Industries, cat. no. DYNJ24720B; includes mask with eye shield, sterile gauze, sterile drapes, an 11‐blade scalpel, small straight scissors, needle driver; as an alternative, individually packaged/sterilized components may be used)
Forceps, e.g., fine‐tipped (Medline Industries, cat. no. MOPAB034)
Sterile gloves (Medline, cat. no. 1075)
Aluminum foil (optional)
Bead sterilizer (Germinator 500, Roboz Surgical Instrument, cat. no. DS‐401)
Sterilization packs (Fisher Scientific, cat. no. 01‐812‐55)
Sterile cotton swabs (Fisher Scientific, cat. no. 23‐400‐124)
Sterile, fine‐tipped marker or pencil
Operative microscope (optional but recommended; Leica Microsystems, cat. no. M651, or similar)
Stereotactic frame (Stoelting, cat. no. 51730D) with mouse stereotaxic gas anesthesia mask (cat. no. 51609M) and warmer control box (cat. no. 53800)
Heating pad for postoperative recovery cage (Sunbeam, cat. no. 863736)
Microinjection unit (Stoelting, cat. no. 53311)
Appropriate sterile attire for the experimenter (e.g., sterile gown, gloves, mask, and protective eyeware)
Anesthetic vaporizer, oxygen supply, induction chamber, and rodent nose cones (Harvard Apparatus, cat. no. 75‐0235), with charcoal filtering canister (AM Bickford, cat. no. 80120)
1‐ml insulin syringes, (Fisher Scientific, cat. no. 14‐829‐1A; for buprenorphine and carprofen injection)
Hair clipper (Wahl, cat. no. 08655)
Surgical drill console (ConMed, cat. no. E9000), with hand piece (ConMed, cat. no. E9010 and cat. no. E9414), and 0.6 mm fine round diamond‐tip drill bit (Stoelting, cat. no. 514552)
50‐µl syringe (Hamilton, cat. no. 7655‐01)
33‐G, 2‐inch, 45° beveled tip, metal removable needle (Hamilton, cat. no. 7803‐05)
Computer for real‐time coordinate calculations (optional but recommended)
4‐0 Vicryl absorbable sutures (Ethicon, cat. no. J494G), or skin closure device per local protocol
Empty animal cage for postoperative recovery

*Monitoring of graft survival and* *in vivo* *bioluminescence imaging*:
d‐Luciferin, powder (MilliporeSigma, cat. no. L9504)
1× phosphate‐buffered saline (PBS; see recipe)

IVIS Spectrum In Vivo Imaging System (PerkinElmer, Revvity)
1‐ml insulin syringes (Fisher Scientific, cat. no. 14‐829‐1A; for luciferin injection)
Living Image Software (IVIS Imaging Systems; PerkinElmer, Revvity)

*CAUTION*: Buprenorphine is a Schedule III controlled substance and should be handled according to appropriate institutional and federal guidelines.

*CAUTION*: Sharp materials should be handled and disposed of appropriately.

#### Section 1: Harvest and prepare cultured stem cells

In this section, we describe cell harvesting procedures for adherent IGF1‐hNSCs and their preparation for injection into 5XFAD or APP/PS1 mice. The amount/concentration of cells can be adjusted for an alternate stem cell type and dosage scheme. However, it is essential to prepare sufficient material to account for inevitable transfer and dead space waste in the injection procedure.

*CAUTION*: Stem cells for transplantation must be handled using sterile solutions and equipment with proper sterile techniques and under a Biosafety Level 2 (BL2) cell culture hood.

*CAUTION*: All waste stem cells and medium must be disposed of appropriately in a disinfecting solution. All biohazardous plastic disposable waste must be disposed appropriately by autoclaving.

1 On the day of the transplantation procedure, remove the T175 flask of IGF1‐hNSCs cultured in growth medium supplemented with 10 ng/ml bFGF from the incubator. Working in the BL2 hood, remove the waste medium and rinse cells with 20 ml of 1× PBS at 37°C. Remove the PBS, add 5 ml of 0.25% trypsin, and incubate at 37.0°C until cells detach.
  Standard operating protocols calls for IGF1‐hNSCs to be cultured on poly‐ *d* ‐lysine‐coated surfaces. We typically use precoated T175 flasks for the present application for convenience and consistency.
  The IGF1‐hNSC cell line was previously stably transduced with firefly luciferase and GFP using the lentivirus vector LV‐Luc2‐P2AEmGFP (Imanis Life Sciences, cat. no. LV050‐L; McGinley et al., 2022).
  IGF1‐hNSC cultures should be ∼70% confluent. One T175 flask will yield ∼5 × 10^7^ IGF1‐hNSCs. However, the number of cells derived from a T175 flask may differ based on stem cell type, cellular characteristics, e.g., size.
  IGF1‐hNSCs can also be obtained directly from frozen stock, but cell viability is likely to be lower than from live cultures.
2 Add 10 ml of 1× trypsin inhibitor, resuspend cells gently, and transfer the cell suspension from each T175 flask into a sterile 15‐ or 50‐ml tube.
3 Centrifuge the cell suspension(s) at 450 ± 20 × *g* (1400 ± 100 rpm) for 5 ± 1 min at room temperature.
4 Without disturbing the cell pellet, carefully remove the supernatant from each tube and gently resuspend each pellet in 14 ml hibernation medium. Cap the tube and mix the suspension by gently inverting the tube two or three times.
5 Allow the cell suspensions in each tube to settle for 2‐3 min. Without disturbing the settled cell debris, transfer no more than 13 ml of cell suspension from each tube into one 50‐ml centrifuge tube. If the total volume to be transferred is >50 ml, divide it equally between two 50‐ml centrifuge tubes.
6 Centrifuge the cell suspension(s) at 450 ± 20 × *g* (1400 ± 100 rpm) for 5 ± 1 min at room temperature.
7 Without disturbing the cell pellet, carefully decant the supernatant from each tube into a sterile waste bottle. Gently resuspend each pellet in 13 ml hibernation medium.
8 Centrifuge the cell suspension at 450 ± 20 × *g* (1400 ± 100 rpm) for 5 ± 1 min at room temperature.
9 Without disturbing the cell pellet, carefully decant the supernatant from the tube into a sterile waste bottle.
10 Combine the cell pellets by resuspension, gently, with 1.8 ml hibernation medium using a 2‐ml pipet. Gently pipet several times to uniformly suspend cells, taking care not to introduce air bubbles, as they shear cells. Transfer the entire cell suspension into a sterile 2‐ml cryovial, and then use a P1000 micropipet to measure the volume to the nearest 0.01 ml. To do so, pipet measured increments of the cell suspension into a new sterile 2‐ml cryovial and record the total volume transferred in the appropriate space in step 12.
11 Count both live and dead cells by standard trypan blue exclusion in a hemocytometer. Calculate cell viability; cells should be transplanted only if their viability is >90%.
  Dead cells and their debris cause inflammation; therefore, >90% cell viability is crucial.
12 Further dilute the cells to their final target concentration with hibernation medium such that the required dose will be administered within 1‐2 µl. Place cells on ice and immediately transport them to the animal procedure area.

#### Section 2: Stereotactic surgery and stem cell transplantation

In this section, we describe how to transplant IGF1‐hNSCs into the fimbria fornix of the hippocampus of 5XFAD or APP/PS1 mice. The process can be subdivided into the preparation of the surgical suite and instruments, surgical preparation (∼10 min duration per mouse), surgical exposure (∼10 min duration per mouse), the injection procedure (∼30 min duration per mouse), and postoperative care (∼30 min duration per mouse). Procedures can be scheduled across multiple days to accommodate the size of the cohort, factoring in animal age at the time of procedure and adjusting the timing of experimental outcome measurements accordingly. Clustering procedures on consecutive days and minimizing the date range over which injections are performed will minimize variability across the cohort.

In this protocol, each animal undergoing transplantation receives six bilateral injections. However, the protocol can be adjusted for an alternate dosage scheme. We have successfully injected between 30,000 and 80,000 cells/µl in 1‐ to 2‐µl volumes per injection site (up to 9.6 × 10^5^ cells per animal). Higher cell concentrations may be optimized but increase the risk of clogging the injection needle. Higher volumes may also be tested but increase the risk of local tissue damage or efflux up the needle tract and out of the brain.

A video guide is also included for reference (Video 1).

**Video 1:** Intracranial stereotactic injection of human neural stem cells in mouse models.

##### Surgical suite and instrument preparation

13 On the day before surgery, administer first mAb immunosuppression dose of CD40 and CD40L antibodies (intraperitoneally [i.p.], 20 mg/kg for each antibody).
14 On the day of surgery, sterilize surgical instruments (forceps, microscissors, syringe, and needle), 0.6‐mm fine round diamond‐tip drill bit, gauze, cotton tip swabs, and aluminum foil (optional) by autoclaving in sterile, single‐use packs or using a bead sterilizer. Wipe down all other equipment (operative microscope, stereotactic frame, heating pad, microinjection unit) and surfaces with 70% (v/v) ethanol.
15 Set up the anesthetic induction chamber. Turn on the integrated mouse heating pad and set it to 37°C. Open the surgical instruments onto a sterile field (e.g., sterilized towel or drape). Cover operative microscope handles, stereotactic frame adjustment knobs, and other frequently handled equipment parts with autoclaved aluminum foil to keep them sterile, as these tools are manipulated intraoperatively.
*CAUTION*: Use of sterile gloves and attire must follow approved institutional protocols and guidelines. It is our practice for the individual performing animal procedures to wear sterile gown and gloves, mask, and protective eyewear for the duration of the procedure to maintain the sterile field. A nonsterile assistant is ideal, though not required, to help maintain sterility and relay injection coordinates throughout the procedure.

###### Surgical preparations

16 Anesthetize the animal in an anesthetic chamber with 5% (v/v) isoflurane in carrier oxygen at a flow rate of ∼1 liter/min.
17 Reduce the isoflurane to ∼1% (v/v) once an adequate plane of anesthesia is attained and place the animal into a nose cone anesthetic delivery system outside of the sterile field.
*IMPORTANT NOTE*: Continuously monitor the animal's respiration rate and reflexes while under anesthesia.
18 Shave the mouse fur between the ears using an electric hair clipper. Avoid contaminating nearby sterile areas/surfaces with hair shavings.
19 Place the mouse in a prone position on the heating pad to maintain body temperature at 37°C throughout the procedure.
*IMPORTANT NOTE*: Monitor mouse body temperature to prevent overheating while under anesthesia, as this can result in respiratory failure.
20 Administer a pre‐surgery dose of buprenorphine analgesic subcutaneously (s.c.), using 0.05‐0.1 mg/kg per mouse, diluted in sterile saline.
*IMPORTANT NOTE*: Buprenorphine at this dose is effective in mice for 8‐12 hr; so, mice should receive the next analgesic, carprofen, within 8 hr (see step 44).
21 Administer a pre‐surgical dose of immunosuppressant, if used (see step 13).
  We take this opportunity to also administer immunosuppressant (the second dose in the initial induction series of immunosuppression, route and dose as above) while the animal is under anesthesia.
22 Secure the animal in the mouthpiece of the stereotaxic frame, and use the ear bars to center the animal's head parallel to the anteroposterior axis of the frame. Secure the ear bars and check that the head is secured by pressing firmly but carefully on the top of the skull.
23 Lubricate the eyes with Puralube Vet Ointment to prevent dryness during anesthesia.
24 Cover the mouse with a new sterile drape, leaving only the head exposed.
25 Disinfect the shaved area with three alternating scrubs of 2% (w/v) chlorhexidine and warm sterile water using cotton swabs.

###### Surgical procedure

**Figure 2:**
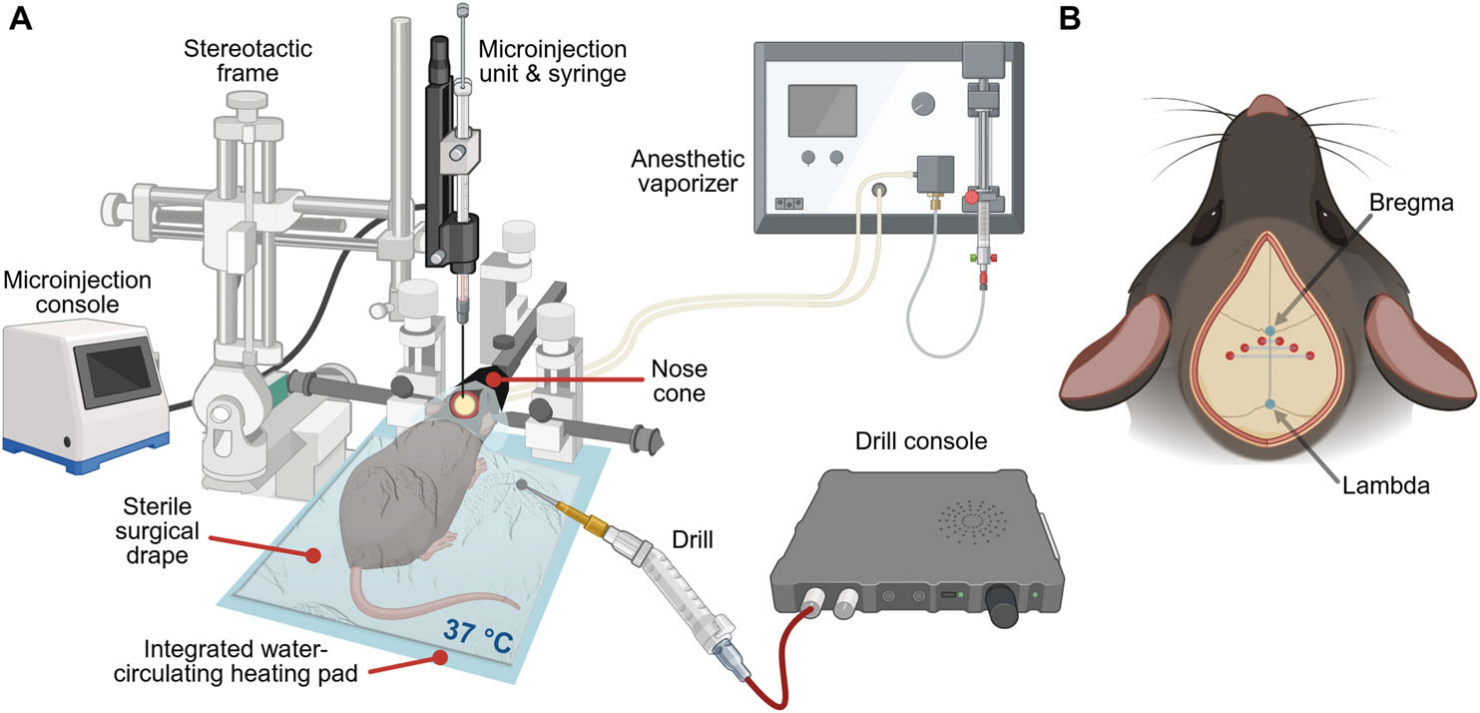
Schematic of the surgical procedure. (**A**) Overall surgical set up, consisting of a stereotactic frame connected to a microinjection unit with a syringe controlled by a console that coordinates are inputted into (see panel B) after calibration to the bregma. A surgical drill handpiece with a 0.6‐mm fine round diamond‐tip drill bit connects to a console. An anesthetic vaporizer with a nose cone keeps the mouse anesthetized, while an integrated water‐circulating heating pad maintains its body temperature. A sterile surgical drape covers the entire mouse, exposing only the surgical field. (**B**) Dorsal view of the mouse skull. Teal circles represent the bregma (top) and lambda (bottom), and red circles represent anteroposterior (A‐P; −0.82/±0.75/−2.5 mm), mediolateral (M‐L; −1.46/±2.3/−2.9 mm), and dorsoventral (D‐V; –1.94/±2.8/−2.9 mm) coordinates relative to the bregma; negative A‐P coordinates indicate locations posterior to the bregma, negative M‐L coordinates indicate locations to the left of midline, and negative D‐V coordinates indicate locations below the bregma‐lambda plane. These red circles represent the *x*‐*y* coordinates that locate the six drill and injection sites within the *x*‐*y* plane; *z* coordinates for each of the six drill and injection sites dictate depth for stem cell injection into the fimbria fornix. A video guide is also included for reference (see Video 1).

26 Make a midline ∼3‐mm incision through the skin between the ears, ranging approximately from the anterior to the posterior attachments of the auricles, to ensure exposure of both the bregma and the lambda (Fig. 2).

27 Carefully dissect through the subcutaneous tissue, and elevate periosteal tissue from the skull with fine‐tipped forceps.
28 Using sterile cotton swabs, gently push the tissues laterally and off of the skull surface until tissues remain separated off the midline and permit adequate exposure.
29 Visually identify the bregma, where the sagittal and coronal sutures intersect. Identify the lambda, where the sagittal and lambdoid sutures intersect.
30 Attach the 50‐µl Hamilton syringe with needle to the microinjection unit. Check that the top plane of the skull is level with the plane of the stereotaxic frame by measuring the bregma and lambda coordinates using the needle tip. Mediolateral (M‐L) and dorsoventral (D‐V) coordinates for the lambda and bregma should be equal. If they are not, adjust the mouse head as necessary and reassess until the difference between M‐L and D‐V coordinates is within ± 0.1 mm as much as possible.
  If possible, a clean but nonsterile assistant should adjust the mouse position to preserve the sterile field. If the surgeon handles the mouse, they must replace their gloves with fresh sterile gloves and may need to re‐drape the mouse.
31 After all adjustments have been made, locate the bregma with the needle. Read out the anteroposterior (A‐P), M‐L, and D‐V coordinates to the assistant, who will input these coordinates into precalculated fields of the injection coordinate file. Coordinates, defined here, are bilateral, and are calculated relative to the bregma reference coordinates (A‐P, M‐L, D‐V): −0.82/±0.75/−2.5, −1.46/±2.3/−2.9, −1.94/±2.8/−2.9 mm (where negative A‐P coordinates indicate posterior to the bregma, negative M‐L coordinates indicate to the left of midline, and negative D‐V coordinates indicate below the bregma‐lambda plane).
32 Mark out the six calculated injection sites directly on the skull using a sterile fine‐tipped marker or pencil.
33 Using a drill with a 0.6‐mm fine round diamond‐tip drill bit, slowly thin the skull at each marked coordinate until the skull is just penetrated and the brain is exposed.
*IMPORTANT NOTE*: Drill very carefully to avoid damaging the brain. Surgical outcomes and survival, as well as assays assessing the effects of IGF1‐hNSCs on disease outcomes, are directly dependent on careful drilling.
34 Place the microinjection unit on a designated sterile area adjacent to the sterile surgical field for the assistant to retrieve. The assistant should detach the syringe and fill it with ∼30 µl of cell suspension (from step 12; IGF1‐hNSC cell‐injected groups) or hibernation medium (vehicle‐injected controls).
*IMPORTANT NOTE*: When not in use, cells should be kept on ice to maintain viability. Before drawing, cells should be gently but thoroughly and evenly resuspended by inversion.
*IMPORTANT NOTE*: Further, cells will start to settle in the syringe intraoperatively. Therefore, the drawn volume and procedure timing must be consistent for all mice to minimize variability in the number of cells administered.
35 The assistant should reattach the syringe to the microinjection unit, wipe it down with a sterile surgical wipe, and return it to the designated sterile area adjacent to the sterile surgical field.
36 Retrieve the microinjection unit and affix it to the stereotaxic frame. Advance the injector plunger until a small droplet is visualized at the needle tip to ensure needle patency.
37 Adjust the frame coordinates to the first injection site and gradually lower the needle to the desired depth.
*IMPORTANT NOTE*: Although the brain may deform slightly, it should quickly reshape as the needle pierces the surface. If the needle deforms the brain severely, stop and replace the needle with a fresh needle.
38 When the needle has reached the target coordinate, administer the desired injection volume at an appropriate flow rate. We use a flow rate of ∼1 µl/min; however, flow rate and volume will depend on the agent being administered.
39 Pause and allow an equilibrium period of ∼1‐2 min before slowly withdrawing the needle to avoid backflow along the needle track.
40 Move the needle to the contralateral injection site. Produce an additional test droplet through the needle to ensure needle patency. Inject according to the above process.
41 Repeat this process for the remaining coordinates until all six injections are completed.
42 Remove the ear bars from the frame and irrigate the surgical site with sterile saline solution.
43 Close the midline incision per protocol. We prefer using an absorbable suture (e.g., Vicryl/polyglactin 910) as this will dissolve and typically does not require additional handling to remove.

###### Postoperative care

44 Transfer the animal to a clean, empty cage and allow it to recover from anesthesia. Use a heat lamp above the cage or a heated water blanket beneath half of the cage to maintain body temperature during recovery. Monitor the animal until it exhibits normal breathing and behavior.
45 Administer the first dose of carprofen (s.c., 5 mg/kg, freshly diluted in sterile saline) within 8 hr of the pre‐surgery buprenorphine dose (in step 20). Carprofen is effective for 24 hr; therefore, repeat one dose every 24 hr until 48 hr after surgery.
46 Monitor animals twice per day for the first 48 hr after surgery and then once daily until 7 days after surgery or until the sutures are removed or dissolved and the incision is healed.
*IMPORTANT NOTE*: Administer additional analgesics if the animal exhibits signs of pain or distress beyond 48 hr. Follow your own institutional guidelines regarding postoperative monitoring.
*IMPORTANT NOTE*: Count both live and dead cells at the end of the procedural day using trypan blue and ensure the cell suspension maintained >90% viability.

#### Section 3: Post‐stem cell transplantation monitoring of graft survival and in vivo bioluminescence imaging

In this section, we describe the CD4‐CD40L antibody immunosuppression dosing schedule and monitoring of graft survival by BLI. A detailed protocol for BLI can be found in the manufacturer documentation (Perkin Elmer).

Cell allograft or autograft may not require immunosuppression.

47 Record mouse body weights weekly for accurate dosing.
48 Starting on the day before surgery, administer mAb immunosuppression doses of CD40 and CD40L antibodies (i.p., 20 mg/kg for each antibody, as in step 13) daily for four consecutive days. After the fourth consecutive dose, administer mAb doses every 7 days until study end.
49 Begin BLI monitoring no earlier than day 2 post surgery to allow the mice 48 hr to recover from surgery. Monitor each mouse over time. Consider using an observer blinded to treatment group to perform BLI and analysis.
50 At the start of each imaging session, initialize the IVIS Spectrum to cool the charge‐coupled device camera to −90°C.
51 Inject mice with d‐luciferin (i.p., 100‐µl injection resuspended at 40 mg/ml in 1× PBS) 10 min before imaging.
52 Anesthetize the mice in an anesthetic chamber with 2% (v/v) isoflurane in carrier oxygen at a flow rate of ∼1 liter/min for ∼6 min before imaging.
53 Once they are adequately anesthetized in the chamber, and 2 min before imaging, transfer mice to the IVIS chamber. Place each mouse in a prone position on a heated imaging platform with their head inserted into a nose cone anesthetic delivery system. Reduce the isoflurane to ∼1.5% (v/v) for the duration of BLI.
54 Run an IVIS scan with the following settings: exposure time 180 s, F/Stop 1, medium pixel binning, field of view C, and subject height 1.50 cm. Select both luminescence and photograph imaging modes, which will generate a quantitative bioluminescence signal (in photons/second) overlaid over a photographic image of the mouse.
  IVIS scan settings may need to be adjusted for variations in this protocol.
55 Analyze IVIS scans in the Living Image Software using automatically generated contour regions of interest with a 10% threshold to eliminate background noise.

## IMMUNOSUPPRESSION BY TACROLIMUS AND MYCOPHENOLATE MOFETIL

In this alternate protocol, we describe immunosuppression with tacrolimus combined with mycophenolate mofetil instead of the CD4‐CD40L antibody regimen in the basic protocol. This alternate tacrolimus‐mycophenolate mofetil protocol is less costly but relatively less effective over the long term, i.e., >1 week, and it requires daily injections versus the weekly injections with the CD4‐CD40L antibody protocol (McGinley et al., 2022). Stress from handling for the daily injections may affect the results of behavioral tests, which is another consideration. However, for short‐term experiments lasting <1 week assessed using outcomes that are unaffected by handling, the tacrolimus‐mycophenolate mofetil protocol may be considered.

*NOTE*: Cell allograft or autograft may not require immunosuppression.

### Additional Materials (also see Basic Protocol)

Mycophenolate mofetil (Genentech, NDC 0004‐0298‐09)
Tacrolimus (Astellas Pharma, product code 301601, NDC 0469‐3016‐01)

***Perform the Basic Protocol as described but replacing the CD4 and CD40L antibody treatments with mycophenolate mofetil and tacrolimus treatments, as follows***:

1 Administer mycophenolate mofetil daily (i.p., 30 mg/kg) starting 1 week pre‐surgery and until 1 week post‐surgery.
2 Administer tacrolimus daily (i.p., 5 mg/kg) starting 1 week pre‐surgery and until the study end.
  Be sure to record mouse body weights weekly for accurate dosing.

## REAGENTS AND SOLUTIONS

### Hibernation medium

1. Measure and add the following to 750 ml deionized water in a 1000‐ml graduated cylinder while mixing with a stir bar (final concentrations in 1000 ml are listed in parentheses):

2.236 g KCl (MilliporeSigma, cat. no. P5405; 30 mM)
0.9 g d‐(+)‐glucose (MilliporeSigma, cat. no. G7021; 5 mM)
0.049 ml MgCl_2_·6H_2_O solution (MilliporeSigma, cat. no. M2393, prepared to 4.9 M; 0.24 mM)
1.314 g NaH_2_PO_4_, anhydrous (MilliporeSigma, cat. no. S5011; 10.95 mM)
0.71g Na_2_HPO_4_ (MilliporeSigma, cat. no. S7907; 5 mM)
3 ml of 60% (w/w) sodium d,l‐lactate solution (MilliporeSigma, cat. no. L7900; 20 mM)

2. Mix thoroughly and adjust the pH to 7.2 using 0.44 g potassium hydroxide (KOH) pellets (≥85% basis; MilliporeSigma, cat. no. P1767).

3. Adjust the solution osmolarity to 320 mOsm by adding 27.0 g d‐sorbitol (Sigma, cat. no. S3889).

4. Bring the volume to 1000 ml with deionized water and filter the solution through a sterile disposable filter unit (1000 ml, 0.2 µm, surfactant‑free cellulose acetate membrane; Nalgene, Thermo Fisher Scientific, cat. no. 161‐0020) under a hood.

5. Date the container and store up to 1 month at 4°C or 1 year at −20°C.

### Phosphate‐buffered solution (PBS), 1×

Dilute 10× PBS solution to 1× in sterile water. Store up to several months at room temperature (20–25°C).

## COMMENTARY

### Background Information

We developed this protocol to reproducibly transplant human IGF1‐hNSCs neural stem cells into mouse hippocampus as a preclinical model for testing cellular therapeutics. The methodology leverages an effective immunosuppressive regimen that supports graft survival of up to 32 weeks post‐transplant. Additionally, graft survival can be monitored *in situ* in live mouse brain through BLI. We have employed this method in two AD mouse models, 5XFAD (Chen et al., 2023; McGinley et al., 2022) and APP/PS1 (McGinley et al., 2018), and find that IGF1‐hNSC transplantation improves disease outcomes (Chen et al., 2023; McGinley et al., 2018).

Our approach can be adapted to alternate disease models, such as Parkinson's disease to replace or support dopaminergic neurons, amyotrophic lateral sclerosis to protect motor neurons and modulate immunity, multiple sclerosis to promote remyelination and modulate immunity, traumatic brain injury (TBI) to enhance recovery and reduce inflammation, and stroke to stimulate neuroregeneration and mitigate damage (Rahimi Darehbagh et al., 2024). Additionally, a spectrum of cellular therapeutics besides neural stem cells may be tested, spanning induced pluripotent stem cells, mesenchymal stem cells, embryonic stem cells, and hematopoietic stem cells (Chen et al., 2024; Rahimi Darehbagh et al., 2024). The outcomes assessed will depend on the disease model: for example, motor skills in Parkinson's disease, amyotrophic lateral sclerosis, stroke, and TBI mouse models, or cognitive outcomes in stroke and TBI.

### Critical Parameters

As a preclinical model for testing potential therapeutics, it is essential to consistently and reproducibility transplant IGF1‐hNSCs to facilitate accurate comparisons across groups. Therefore, it is critical to carefully control factors that influence IGF1‐hNSC viability and counts, and surgical complications.

#### IGF1‐hNSC viability and counts

IGF1‐hNSC viability must be high, >90%, to ensure a high‐quality graft that will be the most likely to exert salutary effects. Dead cells and debris will be ineffective and can be detrimental by triggering microglial inflammation. Therefore, when cells are harvested, care is needed while detaching, pelleting, and resuspending the stem cells to minimize damage. Once harvested, stem cells must be kept at 4°C or on wet ice to maintain viability in between injections. To facilitate comparisons across mice and groups, the same number of IGF1‐hNSC cells must be injected per mouse. For large projects, that will necessitate injecting multiple cohorts on several days; the cell counting step with trypan blue is critical to guarantee consistency between cohorts. On the day of surgeries, it is crucial to ensure that the cell suspension is uniform by gently inverting the syringe, as otherwise fewer cells will be injected over the course of the day as cells settle. IGF1‐hNSCs can also be obtained directly from frozen stock, but the resulting cell viability will likely be lower than from live cultures.

#### Surgical complications

Before surgery, it is essential to set up a sterile surgical field, maintained during the entire procedure. Lack of sterility can trigger surgical complications and/or infections, which can cause neuroinflammation that might abrogate any potential positive effects from the injected IGF1‐hNSC cells. During surgery, it is necessary to check the vital signs of mice undergoing the procedure, confirming breathing and optimal body temperature. These efforts will maximize the success of the surgery and survival and minimize complications that may reduce sample size or otherwise interfere with the effectiveness of IGF1‐hNSC transplant. Perhaps the most critical steps during surgery are drilling and injection. Drilling must be performed slowly and carefully to just penetrate the skull. If the drill damages the brain, the trauma and neural injury will confound the results. There is a similar consideration when injecting the cells: pressure from the needle may distort the brain but should be minimal to avoid trauma and neural injury. A fresh, sharp needle should minimize this potential issue. Finally, suturing and post‐surgery care (e.g., analgesia, monitoring breathing and body temperature) are essential to prevent complications, maximizing survival and minimizing inflammation to prevent confounding results.

### Troubleshooting

For troubleshooting suggestions, see Table 1.

**Table 1:** Troubleshooting Guide for IGF1‐hNSC Transplantation

Problem	Possible cause	Solution
Low viability of IGF1‐hNSC cells	Unhealthy culture	Obtain a new culture and check the quality of cells and for possible contamination, e.g., with mycoplasma
	Cells were enzymatically digested for too long	Reduce digestion time or use a milder detachment method
	Cells were sheared and damaged	Avoid aggressive titration and avoid bubbles while pipetting cells
	Cells died after digestion and harvest	Maintain cells at 4°C after digestion and harvest, and use within reasonable time after harvest (∼8 hr)
Clumping of IGF1‐hNSC cells	Cells were enzymatically digested for too little time	Increase digestion time or use fresh enzymatic digestion solution
	Cells were not filtered through a cell strainer	Filter cells through a strainer
Large variability in transplanted IGF1‐hNSC cells	Cells counted inaccurately	Perform three trypan blue counts
	Cells settling in syringe before injection	Gently invert syringe to resuspend cells before each injection
	Inaccurate injected volume	Check settings on microinjection unit
Graft does not survive	Suboptimal immunosuppression regimen	Optimize immunosuppression regimen
	Immunosuppression antibody cocktail ineffective	Obtain a new lot of antibody cocktail
Graft survives but does not improve outcomes	Surgical suite was not sterile—may cause brain infection	Sterilize the surgical field more effectively
	Drilling is not careful—may cause brain injury	Perform drilling more carefully
	Injection deforms the brain—may cause injury	Use a new needle that pierces the brain will less deformation
	Poor post‐surgical care—may cause brain infection	More carefully monitor mice postoperatively

### Understanding Results

**Figure 3:**
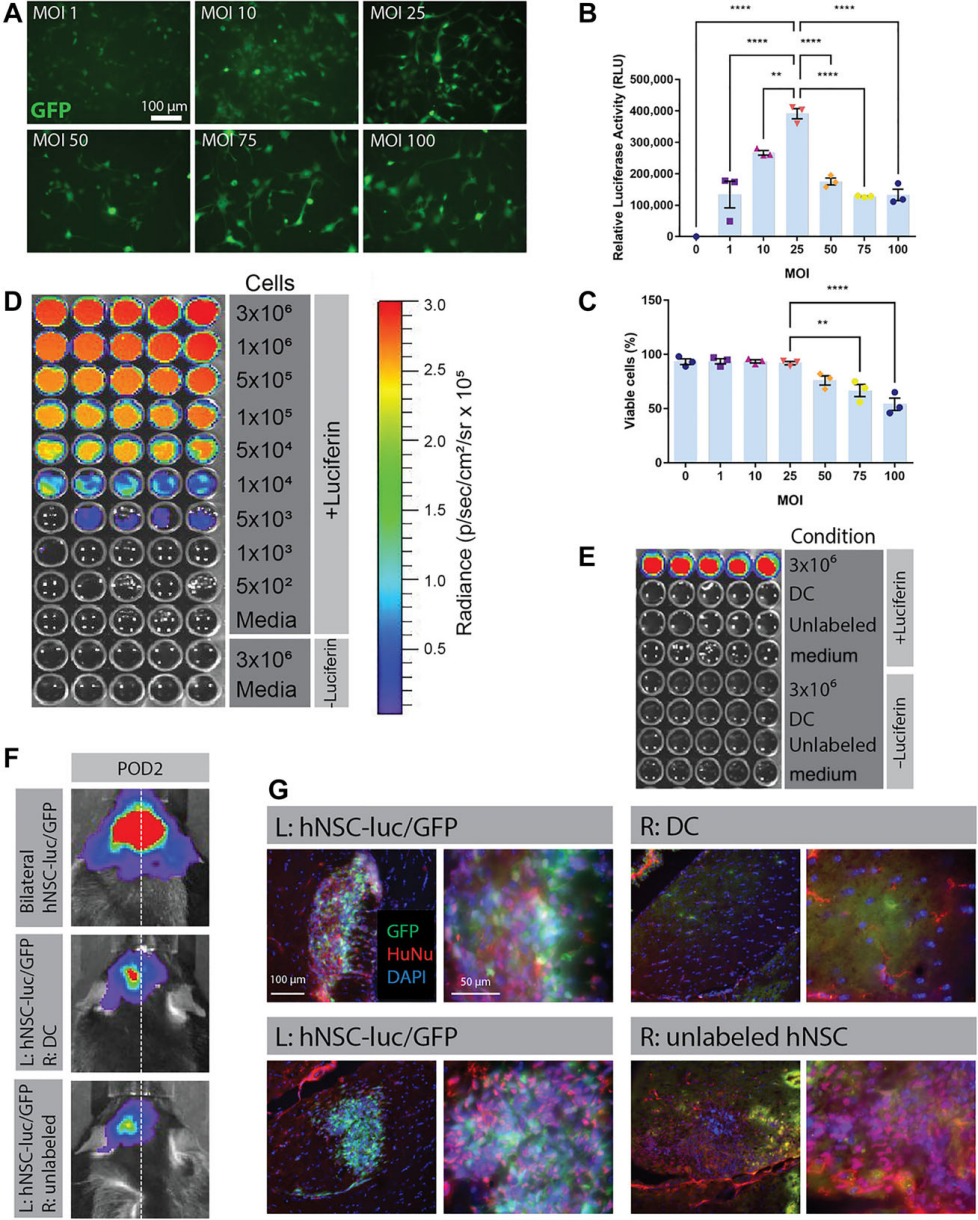
Development and validation of bioluminescent imaging (BLI) to assess transplanted insulin‐like growth factor 1‐expressing human neural stem cell (IGF1‐hNSC) graft viability *in vivo*. (**A**) Fluorescence microscopy of green fluorescent protein (GFP) expression in IGF1‐hNSCs modified to express a dual reporter luc^+^/GFP^+^ vector at increasing multiplicity of infection (MOI) 48 hr post‐transduction. Luciferase assay (**B**) and trypan blue exclusion viability assay (**C**) of IGF1‐hNSC‐luc^+^/GFP^+^ performed 72 hr after transduction. (**D**) *In vitro* BLI of IGF1‐hNSC‐luc^+^/GFP^+^ cells at concentrations ranging from 3 × 10^6^ to 5 × 10^2^ per well, with no‐luciferin and medium‐only controls. (**E**) *In vitro* BLI of unlabeled IGF1‐hNSC and dead IGF1‐hNSC‐luc^+^/GFP^+^ (DC), with IGF1‐hNSC‐luc^+^/GFP^+^ cells as a positive control. (**F**) *In vivo* BLI detection of transplanted hNSC‐luc^+^/GFP^+^ in 8‐week‐old C57BL/6J mice on post‐operative day (POD) 2 after bilateral injection of 3.6 × 10^5^ IGF1‐hNSC‐luc^+^/GFP^+^, or unilateral injection of 1.8 × 10^5^ IGF1‐hNSC‐luc^+^/GFP^+^ (L: left side) with contralateral injection of 1.8 × 10^5^ DC or unlabeled IGF1‐hNSC transplants (*n* = 2 per group). (**G**) Representative POD 2 immunohistochemical images showing the fimbria fornix target area in C57BL/6J mice, with IGF1‐hNSC‐luc^+^/GFP^+^ grafts expressing GFP (green) and human‐specific nuclear antibody HuNu (red), with contralateral staining of DC or unlabeled hNSC. Data presented as mean ± standard error of the mean for luciferase activity and cell viability analyzed by ANOVA with Tukey's post‐test for comparisons of multiple groups; **p < .01; ****p < .0001. Figure from McGinley et al. (2022), https://doi.org/10.1002/ctm2.1046, used under CC BY 4.0 license.

**Figure 4:**
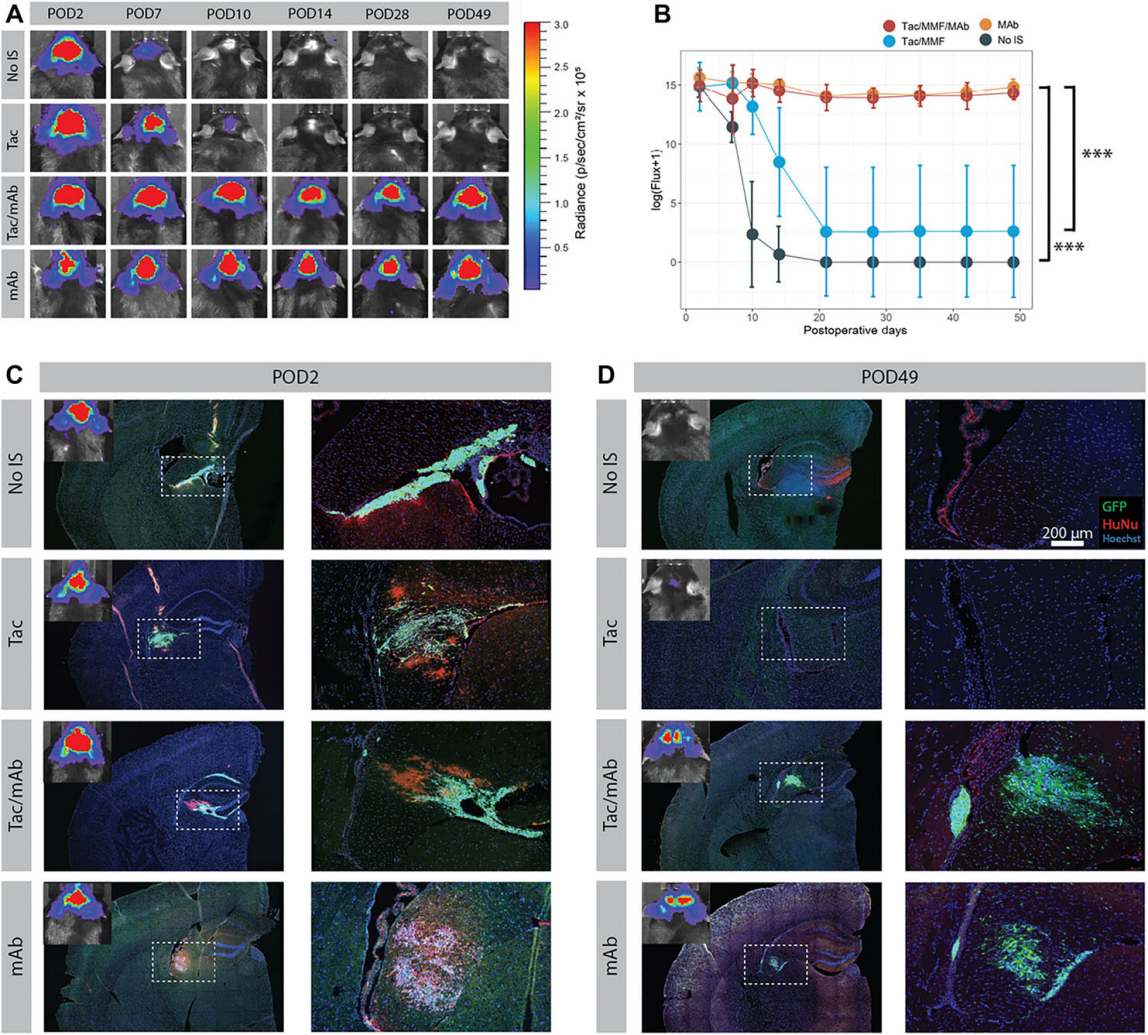
Assessment of immunosuppression protocols using bioluminescent imaging (BLI) to track transplanted insulin‐like growth factor 1‐expressing human neural stem cell (IGF1‐hNSC) graft survival. (**A**) Serial BLI detection of transplanted IGF1‐hNSC‐luc^+^/green fluorescent protein (GFP)^+^ in C57BL/6J mice (3.6 × 10^5^ total cells) receiving no immunosuppression (No IS), tacrolimus with mycophenolate mofetil (Tac/MMF), Tac/MMF in combination with mAbs against CD40L and CD4 (Tac/MMF/mAbs), or only mAbs against CD40L and CD4 (mAb). (**B**) BLI signal quantification for all mice at all timepoints demonstrates significant maintenance of BLI signal in mAb‐treated groups versus groups receiving Tac/MMF alone or non‐immunosuppressed groups. Data presented as mean ± standard deviation for repeated BLI measures, analyzed by linear mixed‐effects model; ***p < .001. (**C and D**) Immunohistochemical (IHC) images showing GFP^+^ grafts (green) colocalized with HuNu (human nuclei; red) in the fimbria fornix target area at post‐operative day (POD) 2 and POD 49 (endpoint). Starting sample sizes: *n* = 10 in No IS group, *n* = 12 in all other groups. Subsets were euthanized for IHC at POD2 (2 from No IS group and 3 from all other groups) and POD10 (2 from No IS group and 3 from all other groups, IHC data not shown). Remaining animals were used for each BLI data point until study end (POD 49). Figure from McGinley et al., https://doi.org/10.1002/ctm2.1046, used under CC BY 4.0 (McGinley et al. 2022).

**Figure 5:**
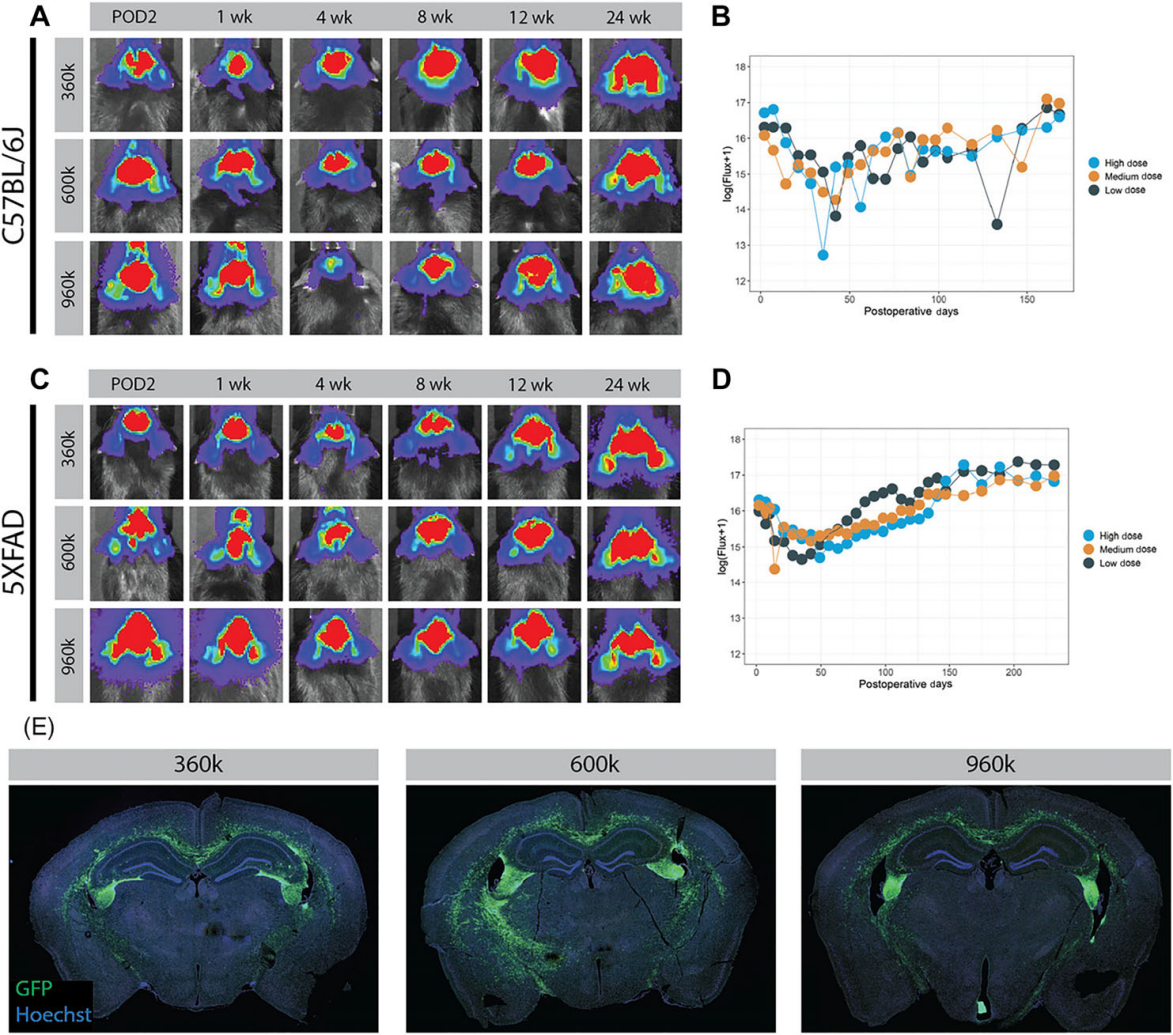
Long‐term bioluminescence imaging (BLI) tracking of transplanted insulin‐like growth factor 1‐expressing human neural stem cells (IGF1‐hNSCs) in C57BL/6J and 5XFAD. Serial BLI detection and signal quantification of 3.6 × 10^5^, 6.0 × 10^5^, or 9.6 × 10^5^ IGF1‐hNSC‐luc^+^/green fluorescent protein (GFP)^+^ cells transplanted in C57BL/6J mice (**A and B**) and 5XFAD Alzheimer's disease model mice (**C and D**) on a dual‐mAb immunosuppression protocol of anti‐CD4 and anti‐CD40L. No biologically relevant statistical differences in BLI flux are seen between cell dose groups. Data for BLI images (C57BL/6J and 5XFAD) and line plot for C57BL/6J mice shown only up to 24 weeks; line plot for graft survival in 5XFAD mice shown up to 32 weeks. (**E**) Representative immunohistochemical images demonstrate GFP^+^ IGF1‐hNSC‐luc^+^/GFP^+^ grafts in the fimbria fornix target area at endpoint in C57BL/6J mice. Sample sizes: *n* = 5 animals per treatment dose (*n* = 15 animals each for C57BL/6J and 5XFAD). Data presented as mean for BLI measures (error bars omitted for clarity), analyzed by linear mixed‐effects model. Supporting Information Figure S1 shows bar plots for POD2, 1 week, 4 weeks, 8 weeks, 12 weeks, and 24 weeks. POD, post‐operative day. Figure from McGinley et al. (2022), https://doi.org/10.1002/ctm2.1046, used under CC BY 4.0 license.

Successful transduction of a target cell line with luciferase is necessary to enable BLI. Titration of viral multiplicity of infection and confirmation of luciferase activity *in vitro* can ensure success when moving to animal models (Fig. 3; McGinley et al., 2022). A benefit of this protocol is confirmation of cell viability with noninvasive BLI. With CD4/CD40L mAb testing, we have seen BLI activity up to 32 weeks post procedure and would expect the cells to continue surviving further with continued immunosuppression. Loss of viability of transplanted cells is evidenced by diminished BLI signal (Fig. 4; McGinley et al., 2022). Besides in vivo BLI, accuracy of injections requires histological analysis to understand the relationship of injected cells with brain anatomy; thus, it is helpful if injected cells carry a fluorescent tag (Fig. 5; McGinley et al., 2022). Alternate methods to identify transplanted cells include source‐specific markers (e.g., human‐specific markers for human‐sourced tissue) or tissue dye placed on the injection needle before penetration of brain tissue.

### Time Considerations

Section 1 of the primary protocol takes ∼1 h. Section 2 of the primary protocol can be subdivided into preparation of the surgical suite and instruments (∼10 min duration), surgical preparations per mouse (∼10 min duration), the surgical exposure per mouse (∼10 min duration), the injection procedure per mouse (∼30 min duration), and closure/postoperative care per mouse (∼30 min duration). Section 3 of the primary protocol will depend on the study design but can range from days to weeks; however, section 3 procedures include immunosuppressant dose preparation (∼10‐30 min depending on cohort size), injections (∼5 min per mouse), and BLI scan (∼20 min for anesthesia and scan for 5 mice).

### Author Contributions

**Kevin S. Chen**: Conceptualization; methodology; investigation; writing—original draft; writing—review and editing; supervision; project administration; funding acquisition. **Kyle J. Loi**: Investigation; writing—review and editing. **Lisa M. McGinley**: Conceptualization; methodology; validation; investigation; supervision. **Osama N. Kashlan**: Conceptualization; methodology; validation; investigation; supervision. **Diana M. Rigan**: Investigation; writing—review and editing. **Shayna N. Mason**: Investigation. **Jacquelin F. Kwentus**: Investigation. **Andrew D. Carter**: Visualization. **Masha G. Savelieff**: Writing—original draft; writing—review and editing; visualization. **Eva L. Feldman**: Writing—review and editing; supervision; project administration; funding acquisition.

### Conflict of Interest

The authors declare no conflicts of interest.

## Supporting information



Supplementary Figure S1. Long‐term bioluminescence imaging (BLI) tracking of transplanted insulin‐like growth factor 1‐expressing human neural stem cells (IGF1‐hNSCs) in C57BL/6J and 5XFAD.

## Data Availability

The data that support the findings of this study are available from the corresponding author upon reasonable request.
