# Supplementary material for: Human Stem Cell Transplantation, Immunosuppression, and Noninvasive In vivo Cell Tracking in the Mouse Brain
Source: Curr Protoc. 2026 Aug 22;6(8):e70442. doi: 10.1002/cpz1.70442 (PMC13499459; doi:10.1002/cpz1.70442)

SUPPLEMENTARY INFORMATION for

Human Stem Cell Transplantation, Immunosuppression, and Noninvasive *In Vivo* Cell Tracking in the Mouse Brain

Kevin S. Chen,[1,2,3,*] Kyle J. Loi,[1,2] Lisa M. McGinley,[1,2] Osama N. Kashlan,[3] Diana M. Rigan,[1,2] Shayna N. Mason,[1,2] Jacquelin F. Kwentus,[1,2] Andrew D. Carter,[1,2] Masha G. Savelieff,[4] Eva L. Feldman[1,2,3]

[1]Department of Neurology, University of Michigan, Ann Arbor, MI, USA

[2]NeuroNetwork for Emerging Therapies, University of Michigan, Ann Arbor, MI, USA

[3]Department of Neurosurgery, University of Michigan, Ann Arbor, MI, USA

[4]Department of Biomedical Sciences, University of North Dakota, Grand Forks, ND, USA

*__Corresponding author__:

Kevin S. Chen, MD. Department of Neurosurgery, University of Michigan, 1500 E. Medical Center Drive, SPC 5338, Ann Arbor, MI, 48109. Phone: 734-936-7010; fax: 734-647-9233; Email: kechen@med.umich.edu.

Supplementary Figure S1. Long-term bioluminescent imaging (BLI) tracking of transplanted insulin-like growth factor 1-expressing human neural stem cells (IGF1-hNSCs) in C57BL/6J and 5XFAD. Serial BLI detection and signal quantification of 3.6×10^5, 6.0×10^5, or 9.6×10^5 IGF1-hNSC-luc+/green fluorescent protein (GFP)+ cells transplanted in C57BL/6J mice (**A**) and 5XFAD Alzheimer's disease mice (**B**) on a dual mAb immunosuppression protocol of anti-CD4 and anti-CD40L. No biologically relevant statistical differences in BLI flux are seen between cell dose groups. Data presented as mean ± standard deviation. POD, post-operative day.

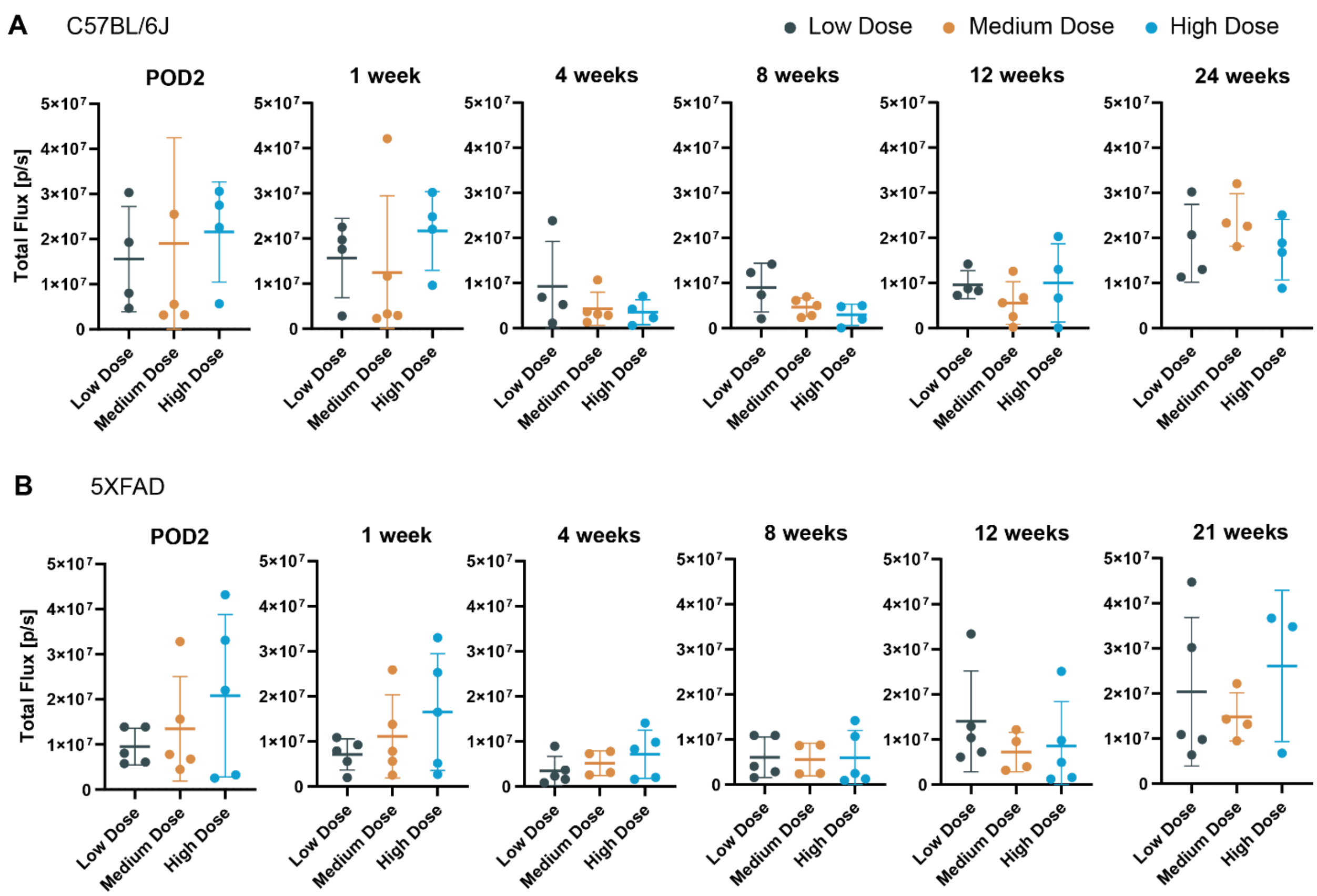

Supplement: Supplementary file 1 [file CPZ1-6-0-s001.pdf]
